# Mitigation strategies for conserving bird diversity under climate change scenarios in Europe: The role of forest naturalization

**DOI:** 10.1371/journal.pone.0202009

**Published:** 2018-08-29

**Authors:** María Martínez-Jauregui, María Jesús Serra-Varela, Mario Díaz, Mario Soliño

**Affiliations:** 1 National Institute for Agriculture and Food Research and Technology (INIA), Forest Research Centre (CIFOR), Madrid, Spain; 2 Sustainable Forest Management Research Institute, University of Valladolid & INIA, Palencia, Spain; 3 Department of Biogeography and Global Change, Museo Nacional de Ciencias Naturales (BGC-MNCN-CSIC), Madrid, Spain; Fred Hutchinson Cancer Research Center, UNITED STATES

## Abstract

There are many possible strategies to promote naturalization in anthropogenic landscapes to mitigate global change effects. We combined large-scale databases available for continental Spain on: (1) distribution of breeding birds, (2) forest inventory stands, (3) land-use cover, (4) 18 global climate models recently developed at local scales, and (5) historical and genetically-based information on the distribution of natural *versus* planted pine forests, to analyze whether back to nature strategies may help to mitigate biodiversity loss due to climate change. We performed the analysis along environmental and ecological gradients of pine forests in Southern Europe. Models suggested that, naturalization strategies, in this case defined by the replacement of planted pine forests and eucalyptus forests by natural pine forests, could help to mitigate the expected loss of bird diversity due to climate change, but that mitigation efficiency will vary along environmental and ecological gradients. Maximum levels of diversity mitigation were predicted at intermediate levels of naturalization, with lower bird richness in areas where all pine forests were either planted or naturalized. Efficiency also varied spatially, given that both cold- and hot-spots of climate-driven bird diversity loss were identified. Transforming planted forest into natural forest is not a mitigation panacea, and additional regionally-adapted strategies may be identified to mitigate the expected biodiversity loss in forest ecosystems.

## Introduction

Climate change is a serious threat to the well-being of humans, and it is considered one of the greatest challenges currently being faced by the world. Climate change is eroding the natural resources and the biodiversity of ecological systems, which will lead to unprecedented consequences unless ambitious mitigation policies are implemented [1,2]. Special attention has recently been given to improving carbon storage, a vital function of different land and marine ecosystems. Among these ecosystems, the relevant role played by forests in contributing to the mitigation of CO2 emissions is regularly emphasized [3]. However, less attention has been paid to the role of forests in mitigating climate-driven biodiversity loss [4–6]. The upcoming decades are expected to bring uncertainty regarding how climate change will have an impact on biodiversity [7,8]. Climate-change impacts on biodiversity must be prevented by developing adaptation and mitigation strategies [9]. Local and regional strategies aimed at improving the ability of species to cope with climate change within their existing range must also be developed [10].

How forests and tree plantations should be managed to make them more complex and thus preserve biodiversity has been largely debated [11,12], mainly under the general “rewilding” or “back to nature” framework of “wilder is better” [13,14]. Here, for the first time, we explore the “back to nature” approach for biodiversity conservation, in this case defined by the natural origin of the forest, within an explicit climate change framework. We specifically attempt to avoid any idealistic view of naturalization as a mitigation tool to prevent climate-driven biodiversity loss and the associated reduction in ecosystem services [15]. The relationships between naturalization and biodiversity are expected, in fact, to be highly variable due to the non-linear responses by species abundance and diversity to climate change [16] and management practices [17].

We explore how bird diversity would change during the process of forest naturalization in continental Spain, a region located in a Mediterranean hotspot [18] where climate change is expected to have major effects on biodiversity [2]. We use bird species richness (an important group to conserve) to analyze the role of forest naturalization as a mitigation strategy for biodiversity conservation under climate change scenarios. Bird species richness has been commonly used as a proxy of biodiversity [19–21]. Further, bird species richness is usually expected to increase as traits associated with “naturalness” or “wildness” increase, although evidence supporting this idea is currently mixed [22–24]. We have taken advantage of results for large-scale long-term monitoring protocols of both pine forests and birds in Spain by combining databases corresponding to: (1) the presence of breeding birds in 3950 10x10-km UTM grid cells [25]; (2) 95,327 forest inventory stands [26,27]; (3) 18 global climate models recently developed at local scales [28]; and (4) historical information and genetically-based information on whether the current pine forests are natural or planted [29]. By modeling all this information, we were able to establish whether forest naturalization has positive, negative or mixed effects on local bird biodiversity, and whether and how this measure could be sufficient for mitigating the expected negative effects of climate change on bird diversity.

## Methods

### Study area

Mediterranean ecosystems are expected to be impacted by climate change due to their position at the trailing edge of species distributions [30]) and to the expected alterations in water regimes, in addition to warming [2,31]. Pine forests in the Mediterranean region are one of the forest systems most intensively managed [32] and occupy a large territory in the Spanish Iberian Peninsula (around 8.5 million hectares in our sample). Five native pine species are distributed across an ecological gradient that ranges from arid lands to temperate forests in the Iberian Peninsula, in the southwestern Mediterranean. Pine forests on the Spanish Iberian Peninsula allow us to investigate the potential role played by forest naturalization on biodiversity at regional scales, and to investigate if naturalization could mitigate the expected diversity loss of forest birds driven by climate change.

To characterize the study area, we estimated a comprehensive list of variables representing the effects of geography, orography, climate, land use, forest composition and structure, and the condition of “planted” versus “natural” with respect to bird diversity (defined by the species richness of pine forest dwelling birds ([24], Table 1). All the variables were calculated on a 10x10-km UTM grid using ArcGIS^®^ software by Esri. The sources used to collect the data were diverse: (1) digital elevation models to calculate orographic variables (25x25 m); (2) the Third Spanish Forest Inventory to characterize forest composition and structure (95,327 permanent sample plots stablished at the intersection of a 1x1 km UTM-grid [26,27]; (3) the Spanish Forest Map (1:50000) to estimate land uses; and (4) historical and genetic data to classify pine forest patches as either planted or natural [29].

**Table 1:** Definition of the variables included in models (Source: [24]).

Code	Variable definitions and levels
**Geographic coordinates**	
*LONG*	Longitude (UTM)
*LAT*	Latitude (UTM)
**Topography**	
*ALTI*	Height above sea level (m): Cell average altitude
*SLOP*	Slope (m): Cell average slope
**Climate**	
*PREC*	Mean annual precipitation (mm)
*TEMP*	Mean annual temperature (°C)
**Land use and forest cover**	
*PINE_COVER*	Pine cover in the cell (ha)
*OCONIF_COVER*	Cover of other conifers in the cell (ha)
*EUC_COVER*	*Eucalytus* spp. cover in the cell (ha)
*OBROADL_COVER*	Other Broadleaves cover (different from Eucalyptus) in the cell (ha)
*SHRUBLAND_COVER*	Shrubland cover in the cell (ha)
*PASTURE_COVER*	Ground and pasture land use cover in the cell (ha)
*AGR_COVER*	Agriculture land use cover in the cell (ha)
*URBAN_COVER*	Urban land use cover in the cell (ha)
*WATER_COVER*	Water land use cover in the cell (ha)
*OTHER_COVER*	Other land use cover in the cell (ha)
**Forest composition and structure**	
*TREE_DIV*	Tree diversity: mean number of different trees available in the forest inventory stands with pine available in the cell
*TREE_DENSITY*	Tree density (n° stems per ha): Tree density average of forest inventory stands with pine available in the cell (trees with a diameter at breast height, dbh> = 7.5cm)
*TREE_BA*	Tree basal area (m^2^ per ha): Mean basal area of forest inventory stands with pine available in the cell (trees with a dbh> = 7.5cm)
*DBH_CV*	Mean coefficient of variation of tree diameter at breast height in forest inventory stands with pine available in the cell. Coefficient of variation of tree diameter at breast height in a stand is calculated as the ratio of the standard deviation to the mean of the diameters of all trees in the stand.
*MONOSP_CH*	Pine monospecific character of the cell: mean of the monospecific character diameter of the forest inventory stands with pine available in the cell. Monospecific character in a stand is defined as the ratio of the pine basal area to total stand basal area.
*TREE_HEIGHT*	Assman dominant height (m): Mean height of the 100 top trees in forest inventory stands with pine available in the cell. 100 top trees in a stand were selected as the 100 trees with the thickest diameter at breast height per hectare.
*SHRUB_DIV*	Shrub diversity: mean number of different shrubs available in the forest inventory stands with pine available in the cell
*SHRUB_CC*	Shrub canopy cover (%): Mean shrub canopy cover of forest inventory stands with pine available in the cell
*SHRUB_HEIGHT*	Shrub height (m): Mean stand shrub height of forest inventory stands with pine available in the cell
***Pinus* plantation**	
*P_PLANT*	*Pinus* forest type: % of the area planted with pine with respect to the total pine forest area in the cell

### Bird diversity in pine forests

We used long-term monitoring of the presence of breeding birds across Spain (particularly 3950 10x10-km cells of the Spanish Breeding Bird Atlas [25] to estimate forest biodiversity based on bird species richness [19], although we are aware that no single indicator could fully capture forest biodiversity [33]. Presence—absence data on the subset of 44 species that are most likely to occupy pine forests (pine forest dwelling birds,) were referenced to the 10x10-km UTM grids throughout Spain. The list of the 44 pine forest dwelling birds was composed by the bird species present in cells where *Pinus* spp. occupied more than 50% of the cell using the Spanish Forest Map (scale 1:50,000), as well as on the information of bird habitat requirements compiled in the bird atlas (see [24] and S1 Table).

### Bioclimatic data and climate scenarios

Local climate models are more appropriate than global scale models such as WORLDCLIM [34] for long-term climate forecasts in the Spanish Iberian Peninsula [35]. Accordingly, we used climate information, including daily precipitation and the maximum and minimum temperatures corresponding to the period 1950–2000, directly obtained from AEMET (Agencia Estatal de Meteorología, Spain). We interpolated 1x1-km climate surfaces corresponding to the mean annual precipitation and the mean annual temperature by means of Thin Plate Splines (TPS) using elevation as the independent co-variable, as it was proposed in [28]. We then used these surfaces to characterize our 10x10-km UTM grid cells by averaging the mean annual precipitation and the mean annual temperature values of all the 1x1 cells included in each grid. The forecasts for these bioclimatic variables representative of 2050 (average for 2041–2060) were obtained from nine of the most recent Global Climate Models (GCMs) used by the Intergovernmental Panel on Climate Change (IPCC) Fifth Assessment Report, from which we selected two different Representative Concentration Pathways (RCP), namely a medium emission scenario (RCP4.5) and a high emission scenario (RCP8.5). The GCMs were transformed to a local scale using the procedure proposed by [28]. We therefore obtained a total of 18 different future climate scenarios (9 GCMs x 2 RCP). The expected bird diversity was estimated for each of these climate scenarios using the model defined in next section.

### Diversity and forest management simulations

The naturalization of forests within the context of this study is defined as a two-step management action. First, current pine plantations, defined as such on the basis of the well-known genetic and historical regions of provenance of pine forests [29], were converted into natural pine forests with other variables (such as the land cover uses or the forest structure) unchanged in the model. Thus simulations take into account a shift in the origin of the forest and their associated bird species, keeping constant the rest of variables influencing local bird species richness (see [24]). Richness of pine forest dwelling birds was calculated at the scale of 10x10-km UTM grid cells for varying proportions of conversion of pine forests from planted to natural. The second step involves the conversion of plantations with exotic trees (mostly eucalypts, *Eucalyptus* spp.) into pine forests, assuming that such exotic plantations do not provide adequate habitat for native birds [36].

We adapted the predictive model proposed by Martínez-Jauregui *et al*. [24] for *Pinus* spp. by taking into account additional interactions between plantation origin, geographical coordinates, and climate variables. All variables used in the model to explain bird diversity were previously inspected to determine linear or curvilinear relationships with respect to the explanatory variable using generalized additive models (“mgcv” library in R 3.3.1), fitting a second-order polynomial transformation when necessary (Table 2). Later, we used a generalized linear model with a normal error distribution and an identity link function, using R 3.1.2 software [37] to estimate pine forest dwelling bird species richness. Main effects of geography, orography, climate, land use, forest composition and structure, the condition of “planted” versus “natural” and interactions between plantation origin, geographical coordinates, and climate variables were used as independent variables. Finally, to cope with multicollinearity issues, we generated a Spearman rank correlation matrix by selecting those variables that were not significantly correlated (rho < |0.7|), and best described the dependent variable. We then calculated the Generalized Variance Inflation Factors (GVIF) for the final models (using the ‘‘car” library in R 3.1.2) to ensure that all GVIF values were below 2.5.

**Table 2:** Model estimates (and standard error) of the variables used to explain pine forest dwelling birds. All variables are standardized; “variable 1: variable 2” represents an interaction between two variables; “poly(variable,2)1” is the linear part of the second-order polynomial fitted for the variable; “poly(variable,2)2” is the quadratic part of the second-order polynomial fitted for the variable. Residual standard error: 4.126 on 3906 degrees of freedom; Adjusted R-squared: 0.6702.

	Estimate	Std.Error	t	value
Intercept	2.161e+01	1.013e-01	213.339	<0.001
poly(*PREC*,2)1	2.997e+01	9.528e+00	3.146	<0.001
poly(*PREC*,2)2	-2.733e+01	5.890e+00	-4.639	<0.001
poly(*TEMP*,2)1	-1.553e+02	8.190e+00	-18.958	<0.001
poly(*TEMP*,2)2	2.042e+01	6.005e+00	3.400	<0.001
poly(*PINE_COVER*,2)1	3.829e+01	7.991e+00	4.792	<0.001
poly(*PINE_COVER*,2)2	-2.864e+01	4.997e+00	-5.731	<0.001
*OBROADL_COVER*	1.066e+00	1.116e-01	9.546	<0.001
*EUC_COVER*	-5.877e-01	9.450e-02	-6.219	<0.001
*SHRUBLAND_COVER*	1.114e-02	9.162e-02	0.122	0.903
*PASTURE_COVER*	1.380e-01	7.635e-02	1.808	0.071
*AGR_COVER*	-2.762e-01	1.165e-01	-2.370	0.018
*URBAN_COVER*	2.892e-01	7.320e-02	3.950	<0.001
*WATER_COVER*	1.637e-01	6.909e-02	2.369	0.018
*OTHER_COVER*	7.501e-02	7.351e-02	1.020	0.307
poly(*TREE_DIV*,2)1	1.172e+02	7.998e+00	14.651	<0.001
poly(*TREE_DIV*,2)2	-5.342e+01	4.835e+00	-11.048	<0.001
*TREE_HEIGHT*	-1.665e-01	1.147e-01	-1.452	0.147
poly(*TREE_BA*,2)1	6.470e+01	1.005e+01	6.434	<0.001
poly(*TREE_BA*,2)2	-2.386e+01	4.981e+00	-4.789	<0.001
*TREE_DENSITY*	-4.015e-01	1.132e-01	-3.547	<0.001
*DBH_CV*	-6.951e-03	8.443e-02	-0.082	0.934
*MONOSP_CH*	-4.579e-01	1.173e-01	-3.902	<0.001
*SHRUB_DIV*	2.141e-01	1.151e-01	1.861	0.063
*SHRUB_HEIGHT*	1.370e-01	8.215e-02	1.667	0.096
*SHRUB_CC*	-1.940e-01	9.191e-02	-2.110	0.035
poly(*P_PLANT*,2)1	-2.081e+01	8.021e+00	-2.594	0.009
poly(*P_PLANT*,2)2	-2.203e+01	5.827e+00	-3.781	<0.001
*LONG*	-1.636e-01	1.298e-01	-1.260	0.208
*LAT*	7.824e-01	1.301e-01	6.015	<0.001
poly(*TEMP*,2)1:poly(*P_PLANT*,2)1	1.457e+02	4.184e+02	0.348	0.728
poly(*TEMP*,2)2:poly(*P_PLANT*,2)1	5.111e+02	3.177e+02	1.608	0.108
poly(*TEMP*,2)1:poly(*P_PLANT*,2)2	-2.148e+02	3.647e+02	-0.589	0.556
poly(*TEMP*,2)2:poly(*P_PLANT*,2)2	-8.458e+02	3.026e+02	-2.796	0.005
poly(*PREC*,2)1:poly(*P_PLANT*,2)1	4.049e+01	5.109e+02	0.079	0.937
poly(*PREC*,2)2:poly(*P_PLANT*,2)1	7.524e+02	3.176e+02	2.369	0.018
poly(*PREC*,2)1:poly(*P_PLANT*,2)2	7.027e+02	5.174e+02	1.358	0.174
poly(*PREC*,2)2:poly(*P_PLANT*,2)2	1.983e+01	3.477e+02	0.057	0.954
poly(*P_PLANT*,2)1: *LONG*	2.463e+01	7.077e+00	3.480	<0.001
poly(*P_PLANT*,2)2: *LONG*	2.962e+01	5.522e+00	5.363	<0.001
poly(*P_PLANT*,2)1: *LAT*	-1.103e+01	7.860e+00	-1.404	0.160
poly(*P_PLANT*,2)2: *LAT*	-1.119e+01	6.475e+00	-1.727	0.084

Once the predictive model was defined, we estimated bird diversity under the uncertainty of different climate scenarios (18 different future climate scenarios explained above) and different naturalization efforts (101 scenarios where the naturalization restriction figure goes from 0% to 100% in every 10x10-km UTM grid cell by an increment of 1% of naturalization). We added a further set of scenarios where the entire surface area occupied by *Eucalyptus* spp. plantations was replaced by native pine forest in order to extend the naturalization process beyond pines to other exotic tree planted on the Iberian Peninsula.

## Results

**Fig 1:**
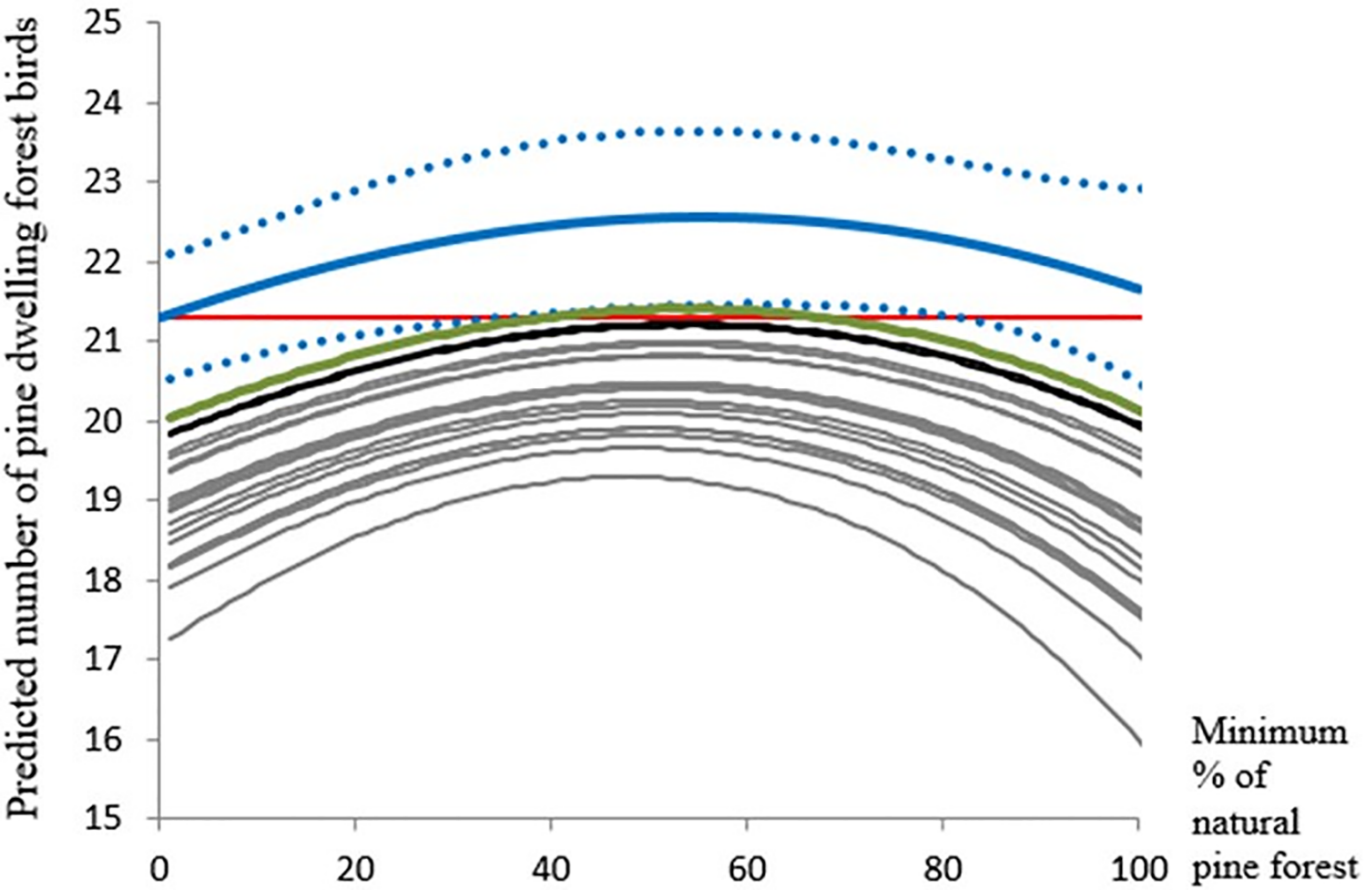
Predicted number of pine dwelling forest birds present in 10x10-km UTM grid cells according to the minimum proportion of natural pine forest under different management and climate scenarios. Red line: current observed mean; blue line: predictions under the current climate and after the first step of the naturalization management action; blue dotted lines indicate the 95% confidence intervals; grey lines: predictions under climate change models and after the first step of the naturalization management action; black lines: predictions under the MRI-CGCM3 global change model (RCP4.5 and RCP8.5) and after the first step of the naturalization management action; green line: predictions under the MRI-CGCM3 global change model (RCP 4.5 and RCP8.5) after the two steps of naturalization.

Topography, climate, land use and forest cover, forest composition, the origin natural *vs* planted pine forest, as well as some interactions between the origin of the forest, geographical coordinates and climatic conditions are important to explain the distribution of bird diversity in the Iberian Peninsula (Table 1). As expected, the number of pine forest dwelling birds would decrease under all the considered climate scenarios (Table 2, Fig 1).

**Fig 2:**
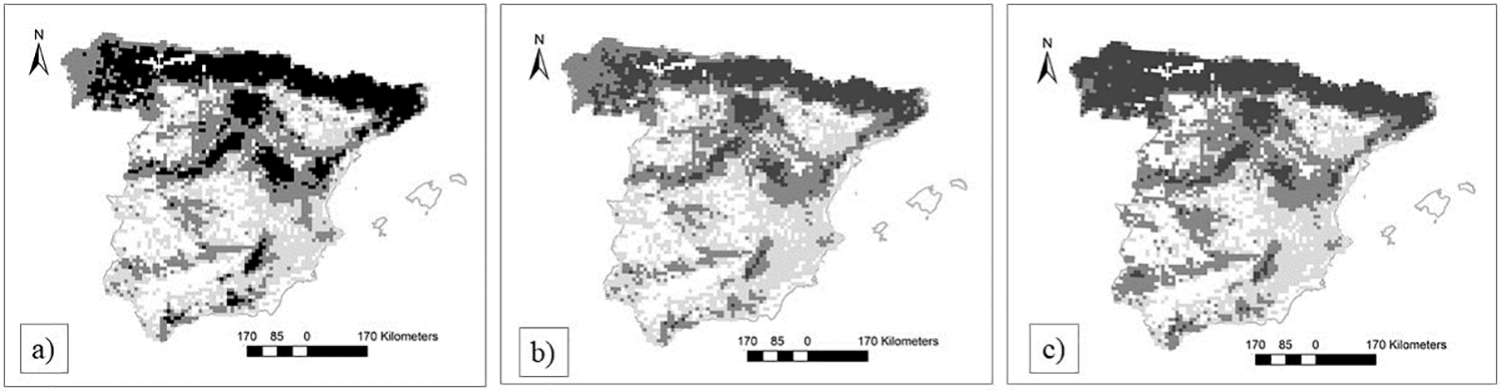
Predicted number of pine dwelling forest birds (a) currently; (b) in 2050 considering climate change (MRI-CGCM3 global change model, RCP4.5) and current management; and (c) in 2050 considering climate change (MRI-CGCM3 global change model, RCP4.5) and naturalization (restriction to approximately 55% natural forests per UTM-cell and the conversion of eucalyptus plantations into pine forests). Grey: < 18 pine dwelling forest birds; dark grey: 18–25 pine dwelling forest birds; black: > 25 pine dwelling forest birds.

**Fig 3:**
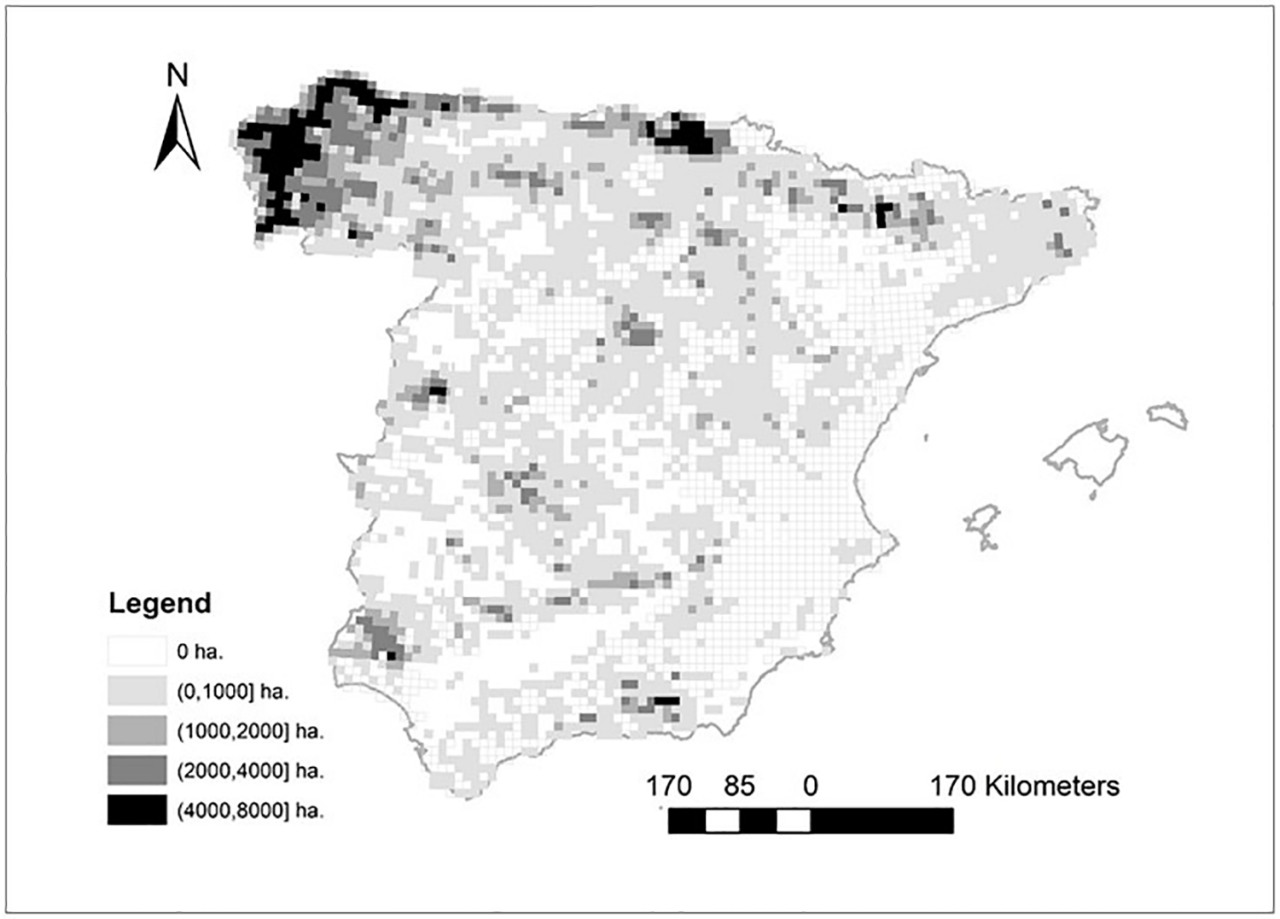
Forest management effort (in hectares) under the MRI-CGCM3 global change model (RCP4.5) and naturalization (restriction to approximately 55% natural forests per UTM-cell and the conversion of eucalyptus plantations into pine forests).

Conversion of pine plantations into natural pine forests and the conversion of eucalypt plantations into natural pine forest can partially mitigate climate-driven biodiversity loss, as natural pine forests usually maintain more bird species than planted ones, and pine forests maintain more species than eucalypt plantations. Nevertheless, the efficiency of the modelled naturalization effort was non-linear, but showed peak positive effects on regional bird richness when around 55% of plantations were transformed to natural pine forests under current climate conditions. Finally, the efficiency and the effort of the modelled naturalization was highly heterogeneous spatially, with higher expected success in the north and in mountains than in the southern lowlands (Figs 2 and 3).

## Discussion

Large-scale combination of biodiversity inventories, information on pine forest origin and structure, climatic variables and land use patterns at regional scales allowed us to analyze whether and how the natural or planted origin of pine forests influenced regional bird diversity [24]. Natural pine forests maintained more species of birds than equivalent pine plantations after correcting for the well-known effects of location, climate, vegetation structure and regional land-use patterns on bird diversity [22,36,38,39]. A possible explanation could be because several differences between planted and natural forests cannot be gathered in our models, such as the scale of structural/age mosaics, the intensified human interventions, the frequency of disturbances, or the removal of dead wood [6,20,40,41]. Moreover these effects of the origin of pine forests on bird diversity vary across the territory. These circumstances suggest possible management tools that could partially mitigate bird diversity loss under climate change.

Here we framed the analyses within the question of whether and how the naturalization of managed forest stands could help to mitigate the expected negative effects of climate change on bird diversity [7–9]. The use of data on bird species richness and predictions from climate change models developed at regional scales allowed analyzing whether naturalization effects would vary geographically under different future climate scenarios.

As expected, we found that the number of pine forest dwelling birds were predicted to decrease under all the considered climate scenarios [42,43]. Losses could be partially offset if planted pine forests were naturalized, as pine forests of natural origin maintain on average more species than their planted counterparts [24]. This could be accomplished by favoring natural regeneration for several tree rotations to facilitate the assembly of a full set of functional groups [23]. However the timeframe to shift a planted pine forest into a natural one needs further investigation.

Nevertheless, the efficiency of the naturalization effort resulted non-linear rather than linear, with peak positive effects at around 55% of plantations naturalized under both current and expected climate conditions. This result suggests that equilibrated proportions of natural and planted pine forests within currently dominant planted *Pine* spp. 10 x 10-km cells may increase the number of species of pine forest dwelling birds [17,24]. Such a result can arise if the list of species linked to plantations is different from the list of natural forests, so that mixes of managed and unmanaged habitat would maintain more species than pure habitat patches [44,45]. Pine plantations are usually inhabited preferentially by early-successional bird species [32], whereas natural forests can maintain preferentially late-successional species more sensitive to human disturbance. Species-specific models would be needed to test this hypothesis, which is consistent with the explanation summarized above to account for the spatial pattern of variation of the effects of pine forest origin on bird diversity [17].

Apart from non-linearity effects, models suggested that conversion of pine plantations into natural pine forests was not enough to fully mitigate climate-driven biodiversity loss. For this reason, we modelled a further additional naturalization effort, consisting in the conversion of eucalypt plantations into natural pine forests. Eucalypt plantations maintain substantially fewer bird species than any other Mediterranean forests due to both its exotic origin and its intensive management [32]. We considered that all of the eucalyptus bird species in Spain were also included in the group of pine dwelling bird species [24]. Despite this restriction, the inclusion of some other species could matter and some limitations can be derived from the list of pine forest dwelling bird species. Therefore, conversion plantations into natural pine forests increased regional bird diversity under all climatic scenarios. However, our results showed that pine naturalization plus eucalypt replacement was able to mitigate fully climate-driven bird diversity loss only under the most favorable scenarios.

Overall, our results indicated that converting all the current planted forests into natural forests would not be the most efficient naturalization strategy to preserve bird diversity under realistic climate change scenarios (with maximum effects at around 55% of plantations naturalized). Further, the efficiency of naturalization would be highly heterogeneous spatially, with well-delimited regions where winners and losers can be identified. Fortunately, local losses would not imply regional extinctions, given that bird distributions will likely expand northwards as bioclimatic conditions change towards conditions now typical from the southwestern Mediterranean region [42,43]. Strategies based on the naturalization of pine and the transformation of eucalypt forests into natural pine forests could better mitigate climate-driven bird diversity loss in the most productive areas in the north and northwest of Spain, than in the south and southwest of Spain, where pine plantations are mostly aimed at restoration rather than production goals due to harsh climate and soil conditions [17,46]. Optimizing the efficiency of naturalization efforts at a regional scale by integrating spatial variability in intensity-biodiversity relationships would mean targeting priority regions and sites for biodiversity enhancement, regions where naturalization would not improve biodiversity conservation, and regions where either forests or plantations could act as dispersal corridors that link current and future suitable habitats [47]. Regional targeting and landscape-scale thinking would then be key to developing wide-scale conservation measures for mitigating biodiversity loss [48].

Therefore, naturalization strategies are useful for mitigating biodiversity loss under climate change scenarios, although non-linear effectiveness and spatial variability should be taken into account before applying such strategies at regional scales. Our analysis was focused on overall pine forest dwelling bird biodiversity rather than considering focal species, and therefore we did not account for the likely roles played by species-specific responses to climate change in terms of demography, dispersal, contemporary evolution, or species interactions [49]. Naturalization strategies should be complemented by considering such species-specific responses and the role of mitigation strategies applied to other global change drivers, such as land-use change and pollution, thereby favoring the preservation of forest biodiversity versus the relentless onslaught of climate change.

## Supporting information

S1 Table Bird species that occupy frequently pine forest for breeding. Total number of 10x10-km UTM grids with presence of Pinus spp and the percentage of pine plantations and natural forest occupied by each species are also shown.
(DOCX)
