# Supplementary material for: Mitigation strategies for conserving bird diversity under climate change scenarios in Europe: The role of forest naturalization
Source: PLoS One. 2018 Aug 29;13(8):e0202009. doi: 10.1371/journal.pone.0202009 (PMC6114515; doi:10.1371/journal.pone.0202009)
Supplement: S1 Table — Total number of 10x10-km UTM grids with presence of Pinus spp and the percentage of pine plantations and natural forest occupied by each species are also shown. (DOCX) [file pone.0202009.s001.docx]

S1 Table. Bird species that occupy frequently pine forest for breeding. Total number of 10x10-km UTM grids with presence of *Pinus* spp and the percentage of pine plantations and natural forest occupied by each species are also shown.

| Scientific name | Number of cells | % Pine plantations | % Natural pine forests |
| --- | --- | --- | --- |
| *Accipiter gentilis* | 1903 | 64,4 | 35,6 |
| *Accipiter nisus* | 2071 | 63,9 | 36,1 |
| *Aegithalos caudatus* | 3212 | 63,8 | 36,2 |
| *Aegolius funereus* | 49 | 4,1 | 95,9 |
| *Aegypius monachus* | 132 | 87,1 | 12,9 |
| *Aquila adalberti* | 129 | 76,0 | 24,0 |
| *Asio otus* | 1027 | 62,4 | 37,6 |
| *Buteo buteo* | 3177 | 69,2 | 30,8 |
| *Carduelis spinus* | 69 | 42,0 | 58,0 |
| *Certhia brachydactyla* | 3262 | 65,0 | 35,0 |
| *Certhia familiaris* | 112 | 59,8 | 40,2 |
| *Circaetus gallicus* | 2253 | 61,4 | 38,6 |
| *Columba palumbus* | 3766 | 64,8 | 35,2 |
| *Cuculus canorus* | 3545 | 64,7 | 35,3 |
| *Dendrocopos major* | 2742 | 68,9 | 31,1 |
| *Dryocopus martius* | 244 | 44,7 | 55,3 |
| *Erithacus rubecula* | 2718 | 67,4 | 32,6 |
| *Ficedula hypoleuca* | 366 | 65,6 | 34,4 |
| *Fringilla coelebs* | 3628 | 66,8 | 33,2 |
| *Garrulus glandarius* | 2940 | 64,6 | 35,4 |
| *Hieraaetus pennatus* | 2090 | 67,5 | 32,5 |
| *Loxia curvirostra* | 1069 | 37,7 | 62,3 |
| *Parus ater* | 2375 | 57,9 | 42,1 |
| *Parus caeruleus* | 3397 | 69,2 | 30,8 |
| *Parus cristatus* | 2359 | 55,4 | 44,6 |
| *Parus major* | 3834 | 65,2 | 34,8 |
| *Pernis apivorus* | 638 | 77,1 | 22,9 |
| *Phylloscopus bonelli* | 2267 | 58,4 | 41,6 |
| *Phylloscopus collybita* | 1165 | 63,0 | 37,0 |
| *Phylloscopus ibericus* | 1794 | 73,8 | 26,2 |
| *Picus viridis* | 3532 | 64,7 | 35,3 |
| *Regulus ignicapilla* | 2164 | 64,0 | 36,0 |
| *Regulus regulus* | 338 | 52,4 | 47,6 |
| *Scolopax rusticola* | 171 | 63,2 | 36,8 |
| *Serinus citrinella* | 370 | 38,1 | 61,9 |
| *Serinus serinus* | 3863 | 65,1 | 34,9 |
| *Sitta europaea* | 1513 | 65,6 | 34,4 |
| *Strix aluco* | 2257 | 64,2 | 35,8 |
| *Tetrao urogallus* | 128 | 35,2 | 64,8 |
| *Troglodytes troglodytes* | 3146 | 67,0 | 33,0 |
| *Turdus merula* | 3869 | 65,3 | 34,7 |
| *Turdus philomelos* | 1485 | 69,0 | 31,0 |
| *Turdus torquatus* | 94 | 17,0 | 83,0 |
| *Turdus viscivorus* | 2871 | 63,4 | 36,6 |
